# Correlation analysis between immune-related genes and cell infiltration revealed prostate cancer immunotherapy biomarkers linked to T cells gamma delta

**DOI:** 10.1038/s41598-023-28475-6

**Published:** 2023-02-11

**Authors:** Wenkang Niu, Tingting Zhang, Lei Ma

**Affiliations:** College of Life Science, Shihezi University, Shihezi, Xinjiang China

**Keywords:** Biomarkers, Prognostic markers

## Abstract

Prostate cancer (PCa) is a urological malignancy with poor prognosis. Immune-related genes are associated with immune infiltration in prostate cancer, but their role in immunogenic PCa is less well understood. We assessed the infiltration patterns of 22 immune cells in PCa and the relationship of immune-related differentially expressed genes (IDEGs) with them. The 87 IDEGs are involved in the interaction between the extracellular matrix and the tumor microenvironment. The model, including seven IDEGs (SLPI, DES, IAPP, NPY, ISG15, PLA2G2A, and HLA-DMB), showed a good predictive power. The SLPI expression is positively correlated with the infiltration level of T cells gamma delta. In addition, PCa has high infiltration levels in Macrophages M1 (18.07%) and Dendritic cells activated (17.64%). The correlation analysis between IDEGs and immune cell infiltration suggested that PCa immunotherapy biomarkers may be closely related to T cells gamma delta.

## Introduction

Prostate cancer (PCa) is a common urological malignancy^1^. PCa, the second most common cancer in male, is the most common type of cancer in 105 countries, especially in Australia, New Zealand, Northern Europe, Western Europe, and the Americas. It is the fifth in terms of mortality^2^. For example, in 2018, there were about 1.276 million cases of PCa worldwide, and about 359,000 patients died of PCa^3^. Some early treatments can improve PCa prognosis and reduce mortality. However, there are still a large number of patients who are resistant to androgen deprivation therapy and become castration-resistant Prostate cancer (CRPC), resulting in shorter survival times^4^.

PCa patients are mainly elderly men with symptoms such as hematuria and dysuria. The detection of serum prostate-specific-antigen was carried out in large numbers since the mid-1990s. PCa has been detected more and more early but often not with any symptoms^5^. Prostate sarcoma also occurs in young people with a low incidence rate and dysuria as the first symptom. However, the disease has a very high degree of malignancy, rapid disease progression and poor prognosis^6^. In the immunosuppressive microenvironment of PCa, tumor cells recruit regulatory T cells and Th17, thereby suppressing antitumor immunity^7^. Higher transforming growth factor-β levels, and lower natural killer cell killing activity, which leads to tumor immunity functional decline^8^. These characteristics determine the limitations of immunotherapy for PCa.

Immunotherapy has emerged as a promising treatment, and its efficacy may be affected by the pattern of immune cell infiltration^9^. For example, the therapeutic cancer vaccine SiPuleucel-T, which is an autologous vaccine, is collected by leukapheresis and then processed by peripheral Dendritic Cells. It is suitable for mCRPC patients with low-volume disease and asymptomatic or mildly symptomatic patients with a slower course of disease, bringing hope for the treatment of PCa patients^10^. There is another checkpoint inhibitor for PCa, ipilimumab, which is a monoclonal antibody against cytotoxic T lymphocyte-associated antigen 4 (CTLA-4). It increases the body's anti-tumor effect by increasing the proliferation and activation of T lymphocytes immune response^11^.

The present study aimed to analyze the correlation between differentially expressed immune-related genes and immune cell infiltration using RNA-seq data from PCa patients and normal controls. Then, immune-related differentially expressed genes (DEGs) were selected by LASSO regression to construct a prognostic model of PCa. Finally, we identified prognostic markers for PCa. These findings may provide new insights for predicting PCa patient survival and personalized therapy.

## Materials and methods

### Data download

The 1793 immune-related genes were obtained from the ImmPort database (https://www.immport.org/). RNA-seq data from PCa patients were downloaded from UCSC Xena database (http://xena.ucsc.edu/), which contained 481 PCa samples and 51 normal samples. Clinical data including overall survival (OS) and survival status were downloaded from the cBioportal Cancer Genomes website (http://www.cbioportal.org/). Clinical data for the Gleason score were downloaded via the 'TCGAbiolinks' R package.

### Screening of DEGs

DEGs between PCa samples and non-tumor tissue samples were screened using the R package 'DESeq2' (|logFC| > 1, FDR < 0.01, P < 0.05). To visualize DEGs, volcano plots were drawn using the R package 'ggplot2'.

### Enrichment analysis of DEGs

Intersections of differentially expressed genes and immune-related genes were used to obtain immune-related differentially expressed genes (IDEGs). Gene Ontology (GO) enrichment analysis and Kyoto Encyclopedia of Genes and Genomes (KEGG) pathway enrichment analysis of IDEGs were performed on the David website (https://david.ncifcrf.gov/) to find out the main biological significance of IDEGs. Significantly enriched (P < 0.05) GO and KEGG pathways were visualized in bar and bubble plots, respectively, using the R package 'ggplot2'.

### Infiltration analysis of immune cells

In this study, the CiberSort algorithm^12^ was used to estimate the proportion of 22 types of immune cell infiltration in 532 samples from all PCa patients. For immune infiltrating cells, the Wilcoxon rank sum test was used to detect the difference (P < 0.05) between PCa samples and normal tissue samples. Using the R package 'ggplot2', a violin plot was drawn to visualize the differences in the infiltration of 22 types of immune cells.

### Correlation analysis between IDEGs and immune cells

The relationship between IDEGs and immune cells was revealed by Spearman correlation analysis. Potential correlations between 22 different immune cell types were analyzed. Then, we analyzed the correlation between OS-related IDEGs and immune cell.

### Construction of prognostic model based on IDEGs

We used LASSO regression analysis of the R package 'glmnet' to determine prognostic markers in PCa. The R package 'Survminer' was used to classify patients with PCa into high-risk and low-risk groups, and define the median risk score. The clinical prognostic power of risk scores was assessed with the R packages 'time ROC' and 'Survminer' using time-related receiver-operating characteristics (ROC) and Kaplan–Meier (K-M) curves. Immunohistochemical sections of IDGEs selected by LASSO regression were downloaded from the Human Protein Atlas database (https://www.proteinatlas.org/).

### Statistical analysis

All statistical analyses were performed using R software (version 4.1.0, http://www.R-project.org). A two-sided P < 0.05 indicated a statistically significant difference. The Wilcoxon rank sum test was used to detect the difference between PCa samples and normal tissue samples. The relationship between IDEGs and immune cells was revealed by Spearman correlation analysis. Kaplan–Meier curves were plotted, and a log-rank test was used to assess the significance of differences in OS.

### Ethics committee approval

The data of this study are from UCSC Xena, ImmPort, cBioportal and HPA database, ethics committee approval was not required due to its exclusive use of public data.

## Results

### Differential expression of IDEGs in PCa tissue and normal tissue

**Figure 1:**
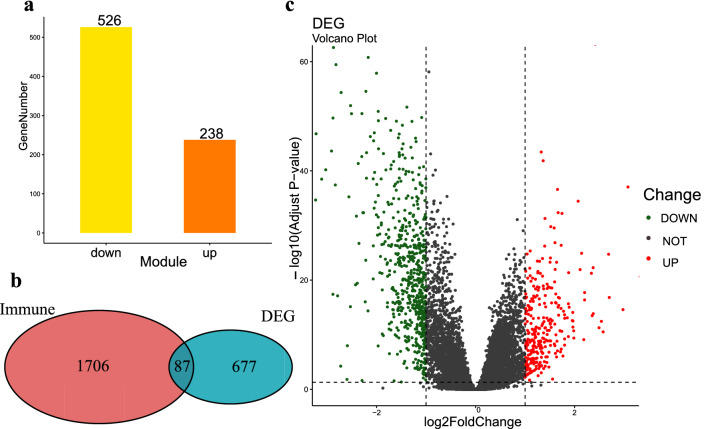
Differential expression of IDEGs in PCa and normal tissue. (**a**) Up-regulated and down regulated differentially expressed genes. (**b**) Veen diagram of differentially expressed genes and immune related genes. (**c**) Volcano map of differentially expressed genes.

By comparing the RNA-seq data of 481 PCa samples and 51 normal samples, we obtained a total of 764 genes (Supplementary Table S1) that were differentially expressed between the cancer and the normal (Fig. 1c), including 238 up-regulated and 526 down-regulated genes (Fig. 1a). After querying the ImmPort database, among these differential genes, 87 genes (Supplementary Table S2) were immune-related genes (Fig. 1b).

### Enrichment analysis of IDEGs

**Figure 2:**
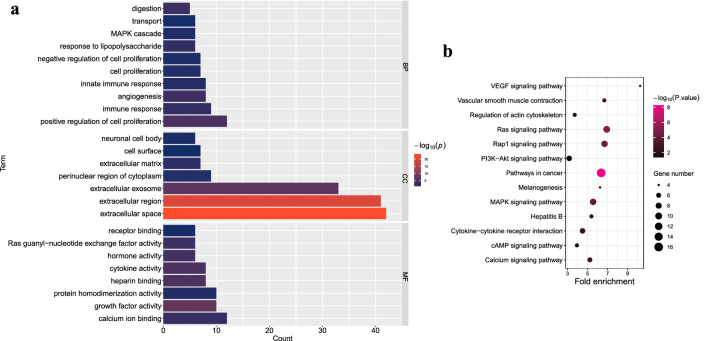
Enrichment analysis of IDEGs. (**a**) Bar chart of the Top 25 go Term. BP, biological processes. CC, cellular components. MF, molecular functions. (**b**) KEGG pathway Term bubble chart.

GO and KEGG enrichment analysis was performed on IDEGs (Fig. 2a,b). Those genes may be involved in various biological processes, such as positive regulation of cell proliferation, innate immune response and negative regulation of cell proliferation. In addition, those genes are also involved in some cellular components, such as extracellular matrix, cell surface and neuronal cell body. Those genes are also involved in some molecular functions, such as calcium ion binding, growth factor activity, cytokine activity and receptor binding. Those genes are involved in some KEGG pathways, such as cytokine-cytokine receptor interaction, PI3K-Akt signaling pathway, cAMP signaling pathway and regulation of actin cytoskeleton. These IDEGs may be associated with the extracellular matrix and cell-to-cell interactions in the tumor microenvironment.

### Correlation analysis between IDEGs and immune cells

**Figure 3:**
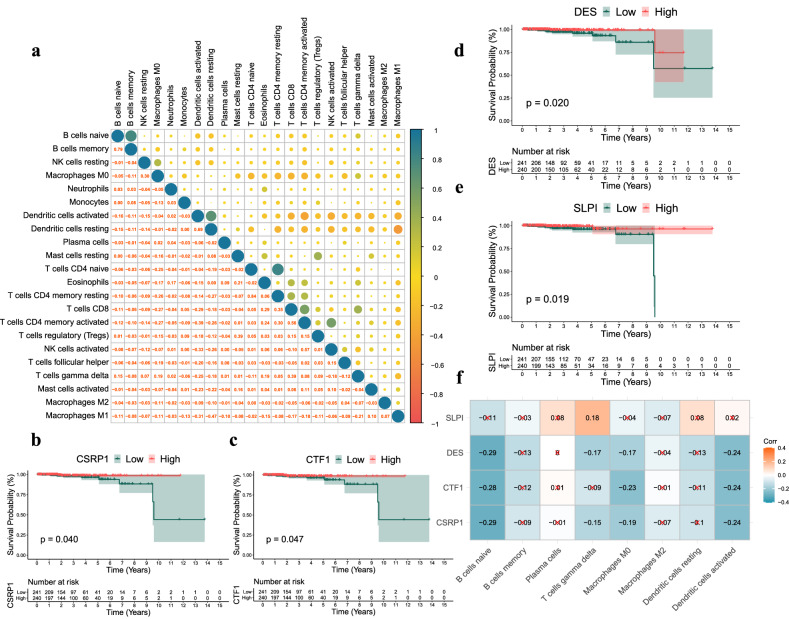
Correlation between IDEGs and immune cells in patients with PCa. (**a**) Correlation between 22 different immune cell types. Blue represents positive correlation and red represents negative correlation. (**b**–**e**) Survival analysis of 4 IDEGs (SLPI, DES, CSRP1 and CTF1). (f) Correlation between IDEGs and immune cells.

To explore the potential interaction between immune cells, we explored potential correlations between 22 different immune cell types (Fig. 3a). Different infiltrating immune cells showed weak to moderate correlations with each other. For example, activated CD4^+^ memory T cells are positively correlated with naive CD4^+^ T cells (r = 0.84), and naive B cells showed a positive correlation with memory B cells (r = 0.79), and Macrophage M1 showed a negative correlation with Dendritic cells resting (r = − 0.47).

Subsequently, we further analyzed the correlation between IDEGs and immune cells. Among 87 IDEGs, 4 IDEGs (SLPI, DES, CSRP1 and CTF1) were significantly associated with OS respectively (P < 0.05) (Fig. 3b–e). Among them, the expression level of SLPI was significantly positively correlated with the infiltration level of T cells gamma delta (r = 0.18), while the expression level of CSRP1 was significantly negatively correlated with the infiltration level of naive B cells (r = − 0.29) (Fig. 3f). The effect of SLPI on T cells gamma delta is that the higher the expression of SLPI, the greater the infiltration level of T cells gamma delta (P < 0.05) (see Supplementary Fig. S1).

These findings suggest that 4 IDEGs, including SLPI, DES, CSRP1, and CTF1, play major regulatory roles in immune infiltrating cells, especially T cells gamma delta.

### Infiltration analysis of immune cells

**Figure 4:**
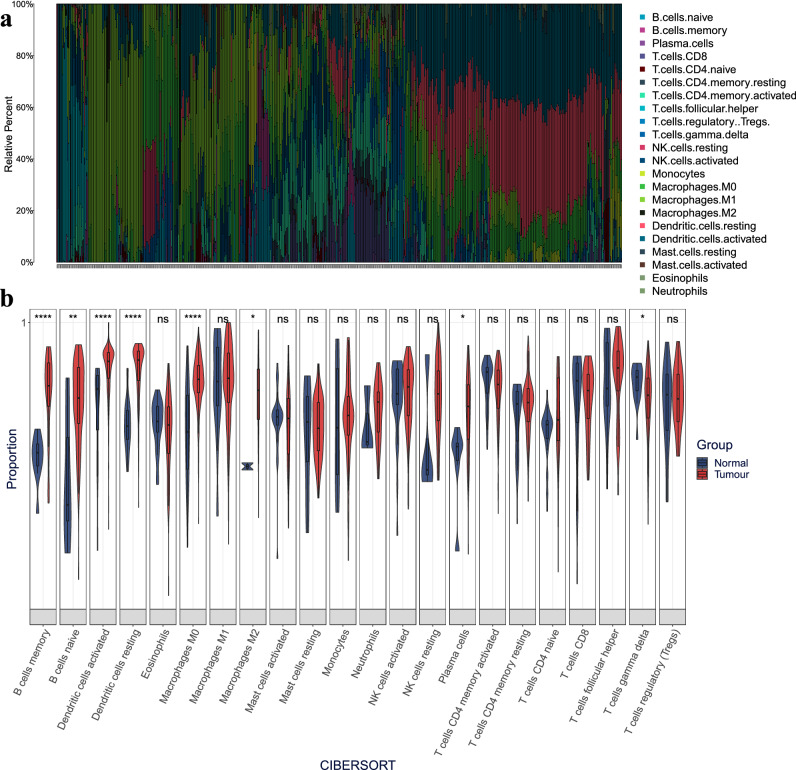
Immune cell infiltration analysis. (**a**) Stacking of the proportion of 22 kinds of immune cell infiltration. The abscissa represents the samples. (**b**) Distribution difference between PCa tissue and normal tissue in 22 kinds of immune cells. *, **, *** and **** indicate P < 0.05, 0.01, 0.001 and 0.0001, respectively. ns: P > 0.05.

In order to explore the infiltration of 22 types of immune cells in PCa tissue, we used the CiberSort algorithm to evaluate the proportion of the infiltration of 22 types of immune cells in each sample (532 in total) (Fig. 4a). The immune cell types with higher infiltration levels were mainly Macrophages M1 (18.07%), Dendritic cells activated (17.64%), Macrophages M0 (14.51%) and Dendritic cells resting (13.57%).

There were differences in the distribution of 22 types of immune cells between PCa tissue and normal tissue (Fig. 4b). For example, the proportion in memory B cells (P = 3.60E−05), naive B cells (P = 3.70E−03), Dendritic cells activated (P = 5.20E−08), Dendritic cells resting (P = 3.00E−12), Macrophages M0 (P = 1.10E−07), M2 macrophages (P = 0.032), and plasma cells (P = 0.016) was higher in normal tissue than in cancer tissue. However, in T cells gamma delta (P = 0.017), the degree in cancer tissue was higher than that in normal tissue.

### Construction of a prognostic risk model for PCa

**Figure 5:**
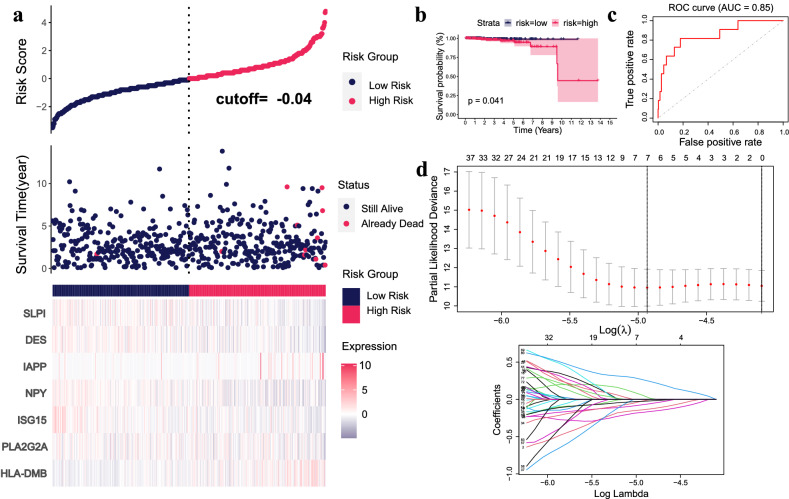
Construction of Prognostic Risk Model for PCa. (**a**) The horizontal axis indicates patients (increasing risk score). The vertical axis indicates risk score (upper), survival time (middle) and genes (lower), respectively. (**b**) Survival curve. Kaplan–Meier for high and low risk groups. (**c**) ROC curve. AUC value of OS prognostic model was 0.85. (**d**) LASSO regression analysis and 10× cross validation.

LASSO regression analysis indicated seven genes of IDEGs, SLPI, DES, IAPP, NPY, ISG15, PLA2G2A and HLA-DMB were associated with survival (Fig. 5d). The risk score is calculated from the expression value of these 7 genes and their Coef values:

$$\begin{aligned} \text{Risk} \, \text{score} & = \left( 0.266929097 \, * \, \text{expression} \, \text{value} \, \text{of }\, \text{HLA-DMB} \right) \hfill \\ & \quad + \left( 0.058427896 \, * \, \text{expression} \, \text{value} \, \text{of } \, \text{IAPP} \right) - \left( 0.175107074 \, * \, \text{expression} \, \text{value} \, \text{of } \, \text{DES} \right) \hfill \\ & \quad - \left( 0.140825333 \, * \, \text{expression} \, \text{value} \, \text{of } \, \text{SLPI} \right) - \left( 0.073003484 \, * \, \text{expression} \, \text{value} \,\text{of } \, \text{NPY} \right) \hfill \\ & \quad - \left( 0.026438393 \, * \, \text{expression} \, \text{value} \, \text{of } \, \text{PLA2G2A} \right) - \left( 0.023222279 \, * \, \text{expression} \, \text{value} \, \text{of } \, \text{ISG15} \right). \hfill \\ \end{aligned}$$

Based on the median risk score, patients were divided into high-risk group and low-risk group. The Kaplan–Meier (K-M) curve showed that the survival rate of the low-risk group was significantly higher than the high-risk group (P = 0.041), indicating that the risk score had a better effect on prediction of the prognosis (Fig. 5b). Risk curves and scatterplots showed risk scores and survival time for all PCa patients (Fig. 5a), and heatmap showed the expression of 7 IDEGs in the high-risk group and the low-risk group (Fig. 5a). The mortality rate and risk coefficient of the low-risk group were lower than that of the high-risk group (Fig. 5b). The AUC value of the OS prognostic model was 0.85 (Fig. 5c).

Overall, the 7 IDEGs had better predictive performance and could be used as prognostic markers for PCa.

### Verification of seven IDEGs

**Figure 6:**
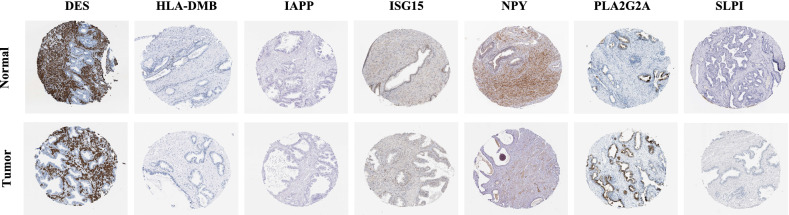
Validation of the differences in expression of the seven genes between PCa and normal tissue at the protein level with data from the HPA database.

Using the Human Protein Atlas database (HPA), we validated the differences in protein expression between PCa tissue and normal tissue (Fig. 6). The immune histochemical sections of the HPA database showed that the protein expression of SLPI, DES, IAPP, NPY, ISG15 and HLA-DMB in normal tissue was higher than that in cancer tissue, while the protein expression of PLA2G2A in normal tissue was lower than that in cancer tissue.

### Correlation between the immune-related gene signature and the clinicopathologic characteristics in PCa patients

**Figure 7:**
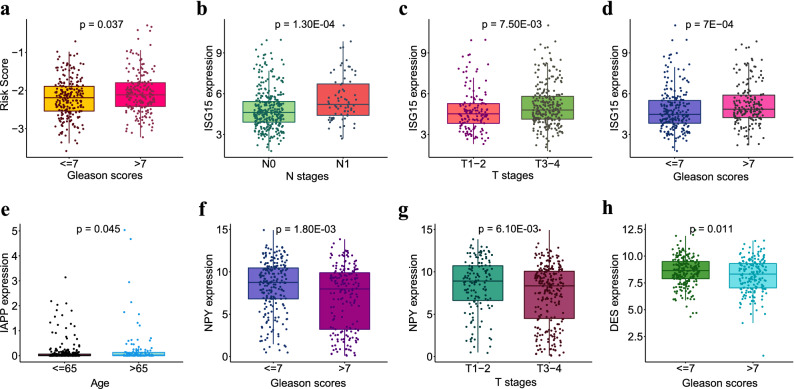
Correlation between the immune-related gene signature and clinicopathologic characteristics in PCa patients. (**a**) Association of the risk score with the response to Gleason scores. (**b**–**d**) Association of the expression of ISG15 gene with T stages, N stages and Gleason scores. (**e**) Association of the expression of IAPP gene with age. (**f**,**g**) Association of the expression of NPY gene with T stages and Gleason scores. (**h**) Association of the expression of DES gene with Gleason scores.

We grouped patients according to age, Tumor Node Metastasis (TNM) stage, and Gleason score, and explored the correlation between these indicators and the 7 IDEGs. After excluding patients with incomplete TNM stage, 402 samples were retained. IDEGs show different expressions in different clinical traits. For example, the expression of ISG15 gene (Fig. 7b–d) was significantly different in different T stages (P = 7.50E−03) and different N stages (P = 1.30E−04). It was significantly different under different Gleason scores (P = 7E−04). In addition, there were significant differences in the expression of IAPP gene (Fig. 7e) in patients of different ages (P = 0.045). There were significant differences in the expression of NPY gene (Fig. 7f,g) in different T stages (P = 6.10E−03) and different Gleason scores (P = 1.80E−03). There were significant differences in the expression of DES gene (Fig. 7h) in different Gleason scores (P = 0.011).

The risk score of the OS-related prediction model in PCa patients was significantly correlated with the Gleason score (Fig. 7a).

## Discussion

In the present study, we established a gene model consisting of 7 IDEGs to monitor the immune status of PCa patients and predict clinical outcomes. The model also showed good clinical predictive capabilities. The AUC value of the model is 0.85. Compared with other reported models, the AUC value and gene numbers of our model are basically consistent. In different cancers, the AUC values of models were 0.622–0.88 across the immune-related genes and autophagy-related genes, containing 3–15 genes^13–17^. Thus, our model is relatively stable. Other studies have used similar algorithms in PCa. Their focus is on the proportion of TIICs and clinical characteristics to assess the risk of PCa recurrence^18^, the relationship between immune cell infiltration and TMB score^19^, use ssGSEA to measure immune infiltration^20^, potential drug target for CRPC, and we screened the same biomarker NPY, which also proved the reliability of our model^21^. But our focus is mainly on the screening of immune-related genes and their correlation with immune infiltrating cells.

### IDEGs function enrichment analysis

These 87 IDEGs have vital functions. These IDEGs are involved in PI3K (phosphatidylinositol-3kinase)-Akt signaling pathway and cAMP signaling pathway. PI3K-Akt signaling pathway is an important signal transduction pathway which is involved in regulating cell proliferation, differentiation, apoptosis and other life activities^22^. The disorder of the PI3K-Akt pathway result in 100% of advanced prostate cancers and 42% of localized prostate cancers. This pathway is a therapeutic target for CPRC clinical trials^23^. In addition, the overexpression of Akt 3 has been confirmed in breast cancer and PCa^24^. Increased intracellular cAMP as a second messenger normally suppresses innate immune functions, including the production of inflammatory mediators and the phagocytosis and killing of microorganisms^25^.

The IDEGs (SLPI, IAPP, NPY, ISG15, PLA2G2A, and HLA-DMB) in the predictive PCa risk model constructed in the present study play an important regulatory role in cancer. Their functions also include immune regulation, inhibition of viral replication and infection, antibacterial, antifungal, antiviral, anti-inflammatory, etc., indicating that our model has good predictive ability. For example, SLPI (Secretory Leukocyte Protease Inhibitor) is a serine protease inhibitor composed of two highly homologous domains, each containing 8 cysteine residues which form 4 disulfide-bonded stabilizing domain structures. The C-terminal region of SLPI has the activity of inhibiting elastase, and the function of its N-terminal region is still unclear. It may have antibacterial, antifungal, antiviral, anti-inflammatory and immunomodulatory activities^26^. SLPI can both fight inflammation, invade pathogens and repair damaged tissue, and its expression level is directly related to the malignancy of cancer cells^27,28^.

Another example, IAPP (Islet Amyloid Polypeptide) is a 37-residue polypeptide, which is one of the main causes of abnormal islet cell function and apoptosis in patients with type II diabetes, and plays an important role in blood sugar control together with insulin^29^. IAPP itself has aggregation, and when incubated with islet cells, it can promote the expression of inflammatory factors and apoptosis^30^. IAPP is similar to other known amyloids such as antibodies and α-synuclein^31^. The dynamic structure and toxicity profile of such amyloid fibrils and plaques suggest that they play an active, long-term role in cellular degeneration and may be therapeutic targets for amyloid diseases^31^. However, IAPP oligomers are the most toxic^31^.

In neuroblastoma, NPY is less processed in tumor tissue, resulting in later clinical staging and poorer prognosis^32^. The expression level of NPY in non-invasive PCa cells was significantly higher than that in invasive PCa cells^33^. Lower NPY expression levels are significantly associated with aggressive clinical behavior of PCa. Thus, NPY may serve as an independent prognostic indicator of PCa progression^33^.

ISG15 (Interferon-Stimulated gene 15) is a member of interferon-stimulated response genes. ISG15 can inhibit virus replication and infection, and is closely related to respiratory virus infection and immune defense mechanisms led by influenza^34^. ISG15 can mediate a variety of antiviral responses, and play an important role in innate immunity and interferon regulatory pathways. Human ISG15 is a key negative regulator of IFN-a/b^35^. ISG15 can also play an antiviral role in immunomodulatory, regulating the host's damage and repairing responses during viral infection. Furthermore, the effects of ISG15 were independent of UbE1L-mediated binding. Although it is not directly antiviral, it limits the expansion of inflammatory responses^36^.

PLA2G2A (Phospholipase A2 group IIA) is a secreted protein whose expression is abnormally increased in pathological states (inflammation, tumor), and may be involved in the development of these pathological changes^37^. In gastric cancer, patients with high PLA2G2A expression have a higher survival rate and a lower incidence of metastasis compared with patients with low expression^38^.

Human leukocyte antigen class II molecules are related to HCV infection^39^, and the HLA class II gene region of HLA-DM gene HLA is divided into HLA-DMA and HLA-DMB^40^. HLA-DM gene polymorphisms are associated with Systemic lupus erythematosus (SLE)^41^, type 1 diabetes (T1D)^42^, etc.

### Immune cell infiltration

The present study evaluated the immune cell infiltration pattern of PCa tissue and normal tissue. We found that the immune cell types with higher infiltration levels in PCa tissue were Macrophages M1 (18.07%), Dendritic cells activated (17.64%), Macrophages M0 (14.51%), and Dendritic cells resting (13.57%). The distribution of Dendritic cells (activated and resting) and Macrophages M0 was significantly different between PCa tissue and normal tissue (P < 0.05). Dendritic cells possess the properties of antigen-presenting cells and have received increasing attention for their possible beneficial roles in lung cancer immunotherapy. Preclinical studies have shown that the administration of Dendritic cells transduced with the chemoattractant CCL21 can increase the infiltration of Dendritic cells, CD4^+^ and CD8^+^ T cells in the lung tumor microenvironment, thereby reducing tumor burden^43^. On the other hand, Macrophages M0 are plastic cells that can change their phenotype under the influence of environmental signals such as radiation damage, and have the potential to migrate to pre-tumor Tumor-associated macrophages (TAMs)^44^. In PCa, TAMs and other myeloid subsets are known to account for 70% of tumor immune subsets, and they influence tumor progression through angiogenesis, tumor cell proliferation, the control of adaptive immunity, and metastasis formation^45^.

We found that T cells gamma delta was associated with the occurrence of PCa patients. T cells gamma delta can rapidly recognize exogenous pathogens and endogenous stress-induced ligands, and initiate adaptive immune responses, which are the first important line of defense in human immune defense and immune surveillance. Among patients enrolled in clinical trials of PCa regimens, the proportion of objective responses to T cells gamma delta immunotherapy was higher than that of standard-of-care second-line therapy (prednisolone, docetaxel combination)^46^. It can directly recognize tumor stress antigens, phosphate compounds and homologous molecules of heat shock proteins, among which phosphate compounds have the ability to specifically expand and activate T cells gamma delta, which has been repeatedly confirmed^47^. T cells gamma delta have been used in adoptive immunotherapy, mainly using phosphoantigens to expand human peripheral blood T cells gamma delta^48^. T cells gamma delta mainly include two cell subsets, Vδ1 T cells and Vδ2 T cells. Vδ1 T cells exert immunosuppressive functions to promote tumor development^49^, Vδ2 T cells are mainly involved in the body's immune surveillance of tumors and defense responses to pathogen invasion^50^. However, there are few studies on the two subsets of T cells gamma delta in PCa. In addition, naive B cells exhibit a significant negative correlation with CSRP1, but the specific mechanism of naive B cells in the pathogenesis of PCa is unclear.

### Relationship between IDGEs and clinical traits

The risk score of the OS-related prediction model in PCa patients was significantly correlated with the Gleason score. It is critical to accurately identify the Gleason pattern 5 (GP5) for prostate adenocarcinoma (PCa) on needle biopsy because the GP5 is associated with disease progression and poorest clinical outcomes^51^. Tumors with a Gleason score ≤ 6 do not develop lymphatic metastasis, and metastatic PCa requires a Gleason score of 4 or 5^52^. Currently, a Gleason score of 8–10 is classified as a high-risk clinical group. For them, all should be treated with the same clinical guidelines, and 2 to 3 years of androgen deprivation therapy should be administered before radiation therapy^53^.

IDGEs are expressed differently in different clinical indicators. In rectal cancer, the extent of peripheral lesions in the T1/T2 group was significantly lower than that in the T3/T4 group^54^. Patients with stage T3/4 in PCa are referred to patients with locally advanced PCa^55^. The spread of cancer cells in lymphatic vessels or lymph nodes can have a significant and similar impact on the prognosis of PCa. Compared with N0, N1 is a more important prognostic parameter^56^. The characteristics of gene expression of IDGEs are closely related to the poor clinical parameters of patients.

## Conclusions

In summary, we assessed the infiltration of 22 types of immune cells in prostate cancer tissues. Meanwhile, some limitations of our study should be acknowledged. We only used the sample of PCa in TCGA, which is a discrepancy discovery from a smaller set of data. With high infiltration degree, Macrophages M1 and Dendritic cells activated play an important role in regulating tumor microenvironment. The seven IDEGs in the PCa prognosis prediction model we constructed can be used as prognostic indicators for PCa. The expression of SLPI gene is positively correlated with the infiltration level of T cells gamma delta. Therefore, the results of this study provide a theoretical basis for predicting the prognosis and survival of PCa patients, and also provide a new target for immunotherapy.

## Supplementary Information

Supplementary Figure S1.

Supplementary Table S1.

Supplementary Table S2.

## Data Availability

Immune-related genes data were downloaded from ImmPort database (https://www.immport.org/shared/genelists), RNA-seq data were downloaded from UCSC Xena database (https://xenabrowser.net/datapages/?dataset=TCGA-PRAD.htseq_counts.tsv&host=https%3A%2F%2Fgdc.xenahubs.net&removeHub=https%3A%2F%2Fxena.treehouse.gi.ucsc.edu%3A443), Clinical data from cBioportal database (https://www.cbioportal.org/study/clinicalData?id=prad_tcga), immunohistochemical sections data were downloaded from HPA database :SLPI gene (https://www.proteinatlas.org/ENSG00000124107-SLPI/tissue/prostate), DES gene (https://www.proteinatlas.org/ENSG00000175084-DES/tissue/prostate), IAPP gene (https://www.proteinatlas.org/ENSG00000121351-IAPP/tissue/prostate), NPY gene (https://www.proteinatlas.org/ENSG00000122585-NPY/tissue/prostate), ISG15 gene (https://www.proteinatlas.org/ENSG00000187608-ISG15/tissue/prostate), PLA2G2A gene (https://www.proteinatlas.org/ENSG00000188257-PLA2G2A/tissue/prostate), HLA-DMB gene (https://www.proteinatlas.org/ENSG00000242574-HLA-DMB/tissue/prostate).
