# Supplementary material for: Correlation analysis between immune-related genes and cell infiltration revealed prostate cancer immunotherapy biomarkers linked to T cells gamma delta
Source: Sci Rep. 2023 Feb 11;13:2459. doi: 10.1038/s41598-023-28475-6 (PMC9922294; doi:10.1038/s41598-023-28475-6)
Supplement: Supplementary file 1 — Supplementary Figure S1. [file 41598_2023_28475_MOESM1_ESM.pdf]

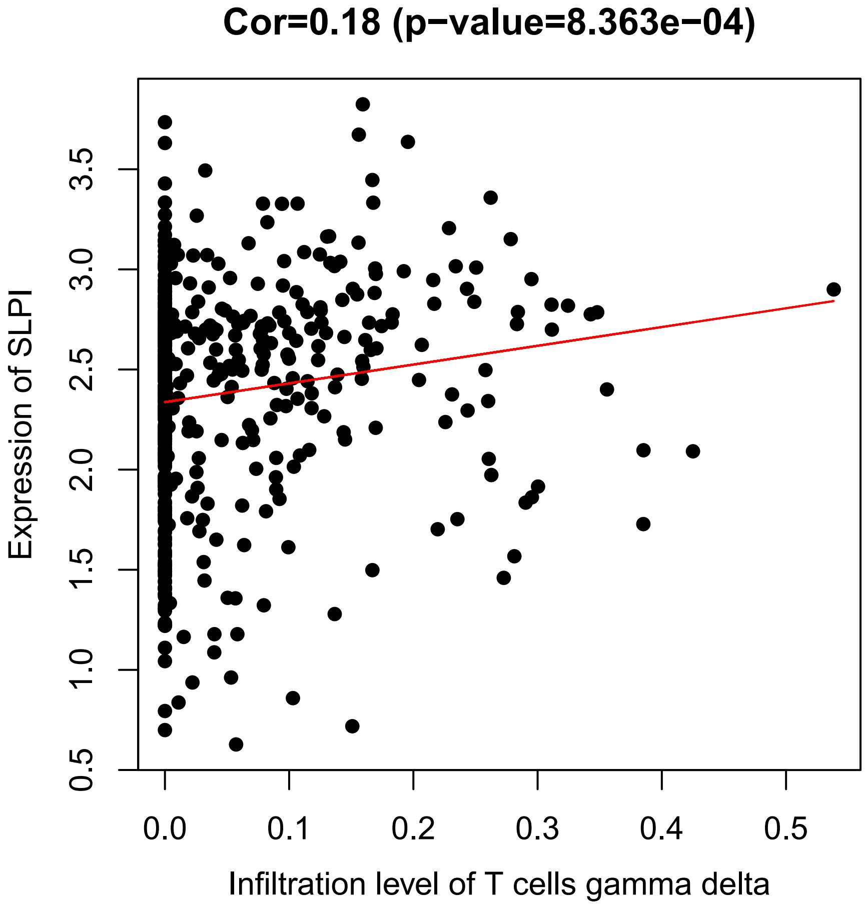

Supplementary Fig. S1 Correlation of SLPI and T cells gamma delta. The horizontal axis represents the infiltration level of T cells gamma delta, and the vertical axis represents the expression of SLPI.
